# The impact of COVID-19 on individual oral health: a scoping review

**DOI:** 10.1186/s12903-022-02463-0

**Published:** 2022-09-22

**Authors:** Virginia Dickson-Swift, Tejashree Kangutkar, Ron Knevel, Sarah Down

**Affiliations:** Violet Vines Marshman Centre for Rural Health Research, Oral Public Health Research Group, La Trobe Rural Health School, La Trobe University, P.O. Box 199, Bendigo, VIC 3552 Australia

**Keywords:** COVID-19, Oral health, Dental health, Oral health care, Dental services

## Abstract

**Background:**

Poor oral health due to dental caries is one of the most prevalent non-communicable diseases worldwide. It has a significant impact on individuals across the lifespan and is a leading cause of preventable hospitalizations. The impacts of COVID-19 on oral health at the practice level are well documented, but gaps in understanding the impact on individual oral health remain. This review addresses this gap.

**Methods:**

Using a JBI scoping review process we mapped and summarized the evidence to identify the impact of COVID-19 on individual oral health. Key search terms were developed, and searches were undertaken by an experienced research librarian.

**Results:**

The 85 included studies were conducted in 23 countries from 5 regions across the world classified using the United Nations Geoscheme system. The majority (82/85) were quantitative, 2 were reviews and there was one qualitative interview study. Cross-sectional surveys were the most common data collection approach followed by an analysis of clinical data, analysis of internet trends and other online methods. Five key areas were identified including changes to the provision of emergency dental services, provision of routine oral health services, oral hygiene maintenance at home, changes in dietary preferences, alternative models of dental provision and help-seeking and attitudes towards dental care in the future.

**Conclusions:**

This scoping review has demonstrated that the pandemic has impacted on oral health at the individual level. It is important that we are aware of these impacts and ensure that support systems are in place to overcome future periods where access to dental care might be compromised. The provision of preventive care remains a vital first step in ensuring good overall oral health as is paramount during periods where access to dental treatment might be limited.

**Supplementary Information:**

The online version contains supplementary material available at 10.1186/s12903-022-02463-0.

## Background

Poor oral health is one of the most prevalent non-communicable diseases (NCDs) worldwide consuming one-fifth of out-of-pocket health expenditure [1–3]. In 2017, it was estimated that oral diseases affect close to 3.5 billion people worldwide, with caries (dental decay) of permanent teeth being the most common condition [4]. It is estimated that 44% of all people worldwide have untreated caries in primary and permanent teeth [5]. International data indicates that dental caries is one of the most prevalent health conditions [6–8] and a leading cause of preventable hospitalization [6]. Poor oral health is also associated with several other chronic diseases including diabetes, stroke and cardiovascular disease [9–12]. Oral health is impacted by a range of social, economic, environmental and political determinants [13] and the impact of COVID-19 on oral health is hypothesized to be significant [14].

The World Health Organization declared the global spread of coronavirus disease (COVID-19) a pandemic on March 11th, 2020 [15]. The impact of COVID-19 across the globe has been significant with more than 6.5 million deaths reported to the WHO to date [16]. A range of measures were implemented to manage the virus including mask-wearing, restrictions on movement, physical distancing, vaccinations, and various forms of lockdowns [17–20]. These measures aimed to contain the virus and limit its impact on health care systems [19]. Throughout the pandemic, healthcare has been considered an essential service however access to some services has been limited or new service models introduced. This has resulted in changes to prevention and treatment services for NCDs (like oral health) and as a consequence low utilisation rate of preventive services have been reported globally [3, 14, 21–24].

The immediate increase in stress and anxiety levels in response to the COVID-19 outbreak has impacted health-promoting behaviors, including oral hygiene [25, 26]. During the pandemic, an increase in prescribing antibiotics and opioid analgesics has been observed in oral health services [27–29]. Significant declines in the utilisation of dental services due to restrictions and regulations on the provision of non-urgent care during the lockdown have also been reported [14, 30, 31]. A recent study in the UK highlighted that reduced access to dental services and cessation of oral health improvement programmes (like supervised toothbrushing) indicate that the COVID-19 pandemic is likely to have a major impact on oral health and result in a widening of inequalities [14]. There is abundant information in the existing literature on the perspectives and experiences of oral health service providers during the COVID-19 pandemic (see, for example, [32–36]). However, information on the oral health impacts of COVID-19 at the individual level remains scant. As the first review in this specific area, our purpose was to undertake a comprehensive mapping and synthesis of the impact of the COVID-19 pandemic on oral health at an individual level.

## Methods

Using a scoping review process our purpose was to explore the breadth or depth of the literature, map and summarize the evidence and identify or address knowledge gaps concerning the impact of COVID-19 on individual oral health [37, 38]. The review was guided by the Joanna Briggs Institute Reviewers’ Manual Methodology for JBI Scoping Reviews [37]. The review question collectively developed by the research team (including oral health and public health researchers and clinicians) was: ‘What is known about the impacts of the COVID-19 pandemic on oral health at the individual level?’.

Inclusion and exclusion criteria, consistent with our review purpose were developed and are outlined in Table 1.

**Table 1:** Inclusion and Exclusion Criteria

Criterion	Inclusion	Exclusion
Population	All individuals including children, adults, vulnerable populations and people with special needs	N/A
Setting	Any	N/A
Interventions	N/A	N/A
Study designs	All study designs	N/A
Publication type	Peer review of original research (including reviews)	Opinion pieces, editorials, magazine articles
Outcomes	Articles that explore the self-perceived oral health impacts of COVID-19 pandemic Articles related to accessing and utilisation of dental services during the COVID-19 pandemic Articles that explore the barriers and enablers of achieving optimal oral health during the COVID-19 pandemic	Articles that explore the transmission of COVID-19 in oral healthcare settings Articles that focus on prevention of COVID-19 at oral healthcare settings Articles that explore the perspectives, experiences and attitudes of oral health service providers on COVID-19 pandemic Articles that focus on challenges or enablers experienced by oral health service providers during the COVID-19 pandemic
Language	Articles written in English	Articles in language other than English
Availability	Full text available	Not full text available
Date	All articles from January 2020 to 10th November 2021	Anything outside of this range

A search strategy was developed in consultation with a specialist health librarian as recommended by the 2020 Methodology for JBI Scoping Reviews and peer-reviewed by using the Peer Review of Electronic Search Strategies (PRESS) checklist [39]. A combination of terms related to two themes of ‘COVID-19 pandemic’ and ‘oral health’ were searched as both, keywords and subject headings (e.g., MeSH) in the titles and abstracts. Boolean operators were used to conduct the search and search limits were applied (see Table 2 for Medline search string). The Preferred Reporting Items of Systematic Reviews extension for scoping reviews (PRISMA-ScR) checklist was used to guide the reporting of the review [40] (see Additional file 1).

**Table 2:** Search String for Medline

Search ID#	Search Terms	Results
S1	Oral Health (MeSH)	18,283
S2	Oral hygiene (MeSH)	13,369
S3	Dental care (MeSH)	22,022
S4	(oral ADJ1 (health OR hygiene OR care))	53,432
S5	Dental health services (MeSH) OR Dental caries (MeSH)	50,988
S6	(dental ADJ1 (health OR hygiene OR care OR caries* OR service* OR practice* OR procedure*))	117,059
S7	Dentists (MeSH)	18,712
S8	Dentist*	134,295
S9	1 OR 2 OR 3 OR 4 OR 5 OR 6 OR 7 OR 8	240,711
S10	COVID-19 (MeSH)	112,182
S11	“covid 19”	179,877
S12	Coronavirus	107,756
S13	“sars cov 2”	117,101
S14	“2019 ncov”	1848
S15	“Severe Acute Respiratory syndrome coronavirus 2”	18,607
S16	10 OR 11 OR 12 OR 13 OR 14 OR 15	200,965
S17	9 AND 16	1325
S18	Limit 17 to English language	1294

The * refers to the truncation used in the MEDLINE search. The * finds all alternate endings to a word

The full search was conducted in Medline, Cumulative Index to Nursing and Allied Health Literature (CINAHL), OVID, Proquest, Embase, Dentistry and Oral Health Sciences Source (DOSS) and Cochrane Database of Systematic Reviews. Search results were then saved and exported into EndNote, a bibliographic software program, to store, organize, and manage all results and then into Covidence [41], part of Cochrane’s systematic review toolkit. After the removal of duplicates, a four-step process was undertaken. One author (TK) independently screened all retrieved articles at the title and abstract stage, with other team members acting as independent second reviewers (VDS, RN, SD). Reviewer disagreements were flagged in Covidence as conflicts and were discussed by the team until consensus was achieved. This process was repeated at the full-text stage, with team discussion occurring where there were differences of opinion. The reference lists of included studies were hand searched and citations of all included studies were checked to ensure search completeness.

A data extraction tool was developed within Covidence and used to extract key characteristics of each included study in tabular format (see Additional file 2). Narrative summaries of the included studies were developed that described how the extracted data related to the aim of the review. Consistent with the guidelines for the effective reporting of scoping reviews [42] and the JBI framework [37] the final stage of the review included a thematic analysis of the key findings of the included studies. Study findings were imported into QSR NVivo with the coding of each line of text. These descriptive codes reflect the key aspects of the included studies related to the impact of COVID-19 at the individual level.

A preliminary protocol for this review was published in the Open Science Framework (OSF-https://osf.io/7t9bq/) preregistrations to enhance transparency and replicability and to reduce any publication or reporting biases.

## Results

**Fig. 1:**
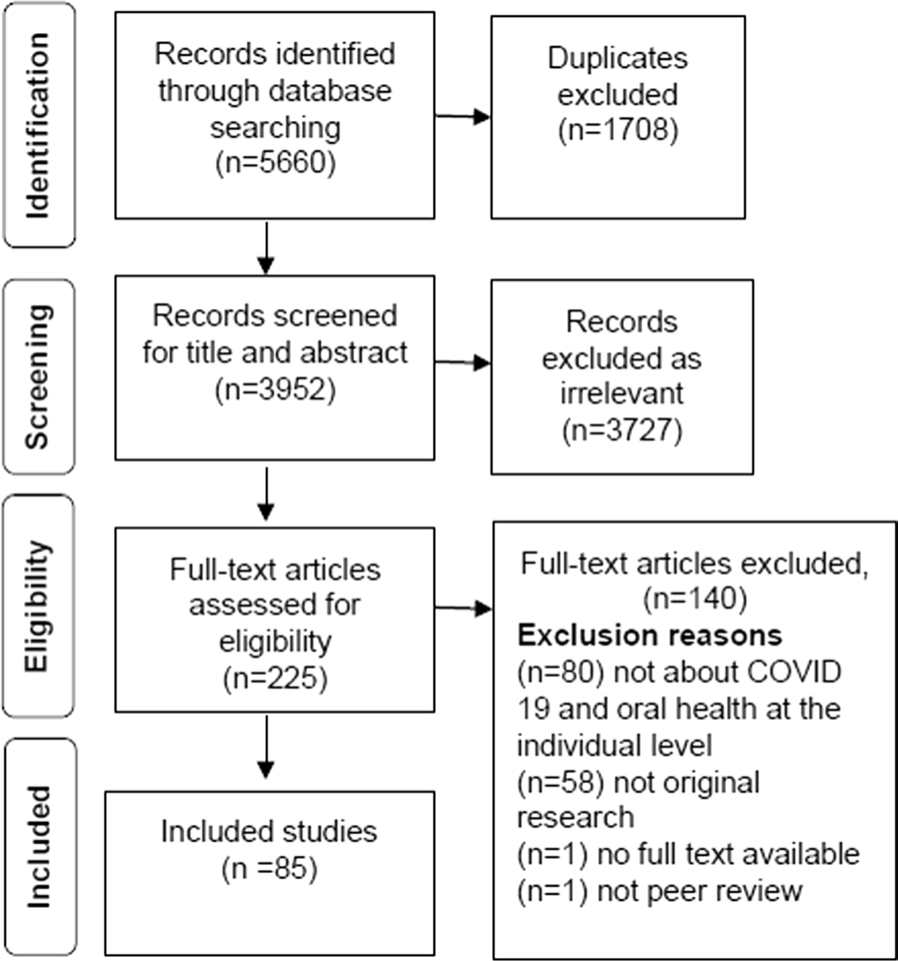
PRISMA flowchart

The search results for this review are outlined in Fig. 1.

A total of 85 studies were included in the review. The United Nations Geoscheme system was used to classify the studies into regional groupings [43]. The studies were undertaken in the following regions: Americas, n = 17, Asia n = 38, Europe n = 25, Oceania n = 1. The highest number of studies were carried out in Brazil (n = 9), Turkey (n = 8) and China (n = 8). Two studies used international samples and 3 studies did not mention a specific geographic region. There were no studies located in Africa or Antarctica.

Data collection methods for the included studies varied with quantitative methods being used most frequently (n = 82). Table 3 provides an overview of the data collection tools.

**Table 3:** Data collection methods of included studies

Data collection method	Number of studies
Questionnaire/survey	42
Clinical records/logbooks/telephone protocols	33
Google/Twitter trends	7
Scoping/systematic review	2
Interview	1

Our synthesis of the key findings of the included studies resulted in the development of the following themes: changes to the provision of emergency dental services, provision of routine oral health services, oral hygiene maintenance at home, changes in dietary preferences, alternative models of dental provision and help-seeking and attitudes to dental care in the future. These themes are reported below. The full dataset of the included studies is provided in Additional File 2.

### Provision and utilisation of emergency oral health services

Most studies concluded that there was an increase in the provision and utilisation of emergency oral health services [44–60]. The key reasons for emergency visits included trauma, acute pulpitis, acute periodontitis and plaque-induced gingivitis/oral mucosal conditions [44, 59]. Studies reporting increases in emergency dental treatment included treatment for dental abscesses [49], temporary fillings [47], tooth extractions [60], antibiotic prescriptions [61], paediatric dental services [59] and orthodontic emergencies [53]. A study undertaken in Saudi Arabia reported that patients were afraid to seek dental care for life-threatening emergencies such as cellulitis during the pandemic [62]. There were some declines in the use of emergency services during the pandemic [62–70] with one study reporting that patients with significant comorbidities sought emergency dental services less frequently during the lockdown period [68].

### Provision and utilisation of preventive and routine oral health services

Most studies reported that there was a decline in the provision and utilisation of routine oral health services [47–49, 52, 60, 64, 69, 71–91] with many studies reporting difficulties accessing oral health services (particularly during lockdown periods). One US study reported that 62% of adults reported delaying dental care due to the COVID-19 pandemic during the spring of 2020 [76]. In Brazil (a country that was hit hard in the first wave) dental procedures decreased from 47 million in the first part of 2019 to 15 million in 2020, representing an overall decrease of about 66% [82]. Similar declines were reported in an Australian study focusing on paediatric dental care [31]. Key barriers identified for not utilising routine oral healthcare services included fear of COVID-19 transmission, lack of oral health symptoms and high treatment costs. Routine oral health care for older dependant adults were affected by lockdowns with a significant reduction (81.14%) reported in one study [73]. Increases in the utilisation of preventive oral health interventions [59] were also reported in one study. Increases in tooth grinding and teeth clenching were reported in an analysis of Google trends internationally [92].

### Oral hygiene maintenance at home

Self-medication or using home remedies for oral health issues were reported [93] with one study finding that parents medicated children with previously prescribed analgesics. Home remedies for orthodontic concerns like loose brackets, bands and loose archwires were also common [94]. Some studies reported that the frequency of oral hygiene maintenance at home increased [77], whilst others reported a decrease [90] or no change. One study reported that women had better oral hygiene behaviours than men throughout the pandemic [95]. The impact of the pandemic on toothbrushing frequency varied [96, 97] with one study from Wuhan showing that children brushed their teeth more during the pandemic [98], whilst another found that parents cared less for their children’s teeth during the pandemic [99]. Toothbrushing frequency was associated with the prevalence of oral problems people encountered [100] and those who had dental prostheses increased prosthesis cleaning during the pandemic [83]. Associations between the frequency of prosthesis and dental cleaning and improved Oral Health Quality of Life (OHRQoL) were also reported [83].

### Changes in dietary preferences

The included studies show mixed results regarding the changes in dietary habits of individuals during the pandemic. Three studies reported an increase in sugary food consumption and the number of meals [96, 97, 101] with increases in consumption of sugars in the form of sweets, jam, honey, and molasses reported in a study from Iran [97]. One study showed that sugar consumption did not change significantly [90]. Very few studies reported that there was a positive change in nutritional status however one study reported that the consumption of sugar-sweetened, flavoured milk and juices, candy and chocolates reduced during the pandemic [97].

### Alternative models of dental provision and online help-seeking

There was increased use of and satisfaction with teledentistry among dental patients during the pandemic [45, 68, 71, 75, 102–104]. A study by HEC da Silva, GNM Santos, AF Leite, CRM Mesquita, PT de Souza Figueiredo, PED Dos Reis, CM Stefani and NS de Melo [104] showed that 78% of patients preferred teledentistry and 92% patients would recommend the use of video consultations to other patients. It was reported that patients strongly agreed and were satisfied with teledentistry in 5 domains: the ease, comprehensiveness and helpfulness of the video consultations and the anxiety and satisfaction levels of the patient [103]. It was also reported that 70% of patients strongly agreed that the video consultations ran smoothly and 75.7% strongly agreed that they were comfortable accessing oral health services from home rather than travelling for the consultations [103]. The use of teledentistry for monitoring cancer in the oral, head and neck regions were well accepted by the patients and improved their quality of life [104]. Tele-dentistry was useful in assessing paediatric dental emergencies. 460 patients accessed the paediatric dental emergency services via telephone during the pandemic [102].

There was also an increase reported in the use of online searches and social media to address/express oral health-related concerns during the pandemic [62, 105–109]. Two studies explored key search terms utilised by those searching online with results showing that “toothache” and “corona-toothache” were used most frequently [105, 109]. One study focused on the use of Twitter throughout the pandemic with people tweeting about oral health impact, types of dental problems, managing symptoms at home, views on consequences of delaying dental treatment and experiences with accessing oral health services [106]. A similar study carried out using the Weibo platform found 12,603 posts related to toothache with 38.9% posts indicating that treatment for dental pain was affected by COVID-19 with many indicating that they couldn’t visit their dentist due to clinic closures [108]. Searches for “teledentistry” and “PPE were also popular [45]. One study showed that tweets related to dental needs were higher in 2020 as compared to 2019 whereas dental advice-related tweets were lower in 2020 as compared to 2019 [110].

### Attitudes towards seeking oral healthcare in the future

Most studies reported that there was a negative impact on the attitudes of individuals towards oral healthcare in the future [54, 58, 62, 68, 77, 95, 99, 100, 106, 111–118]. In one study, 41% of patients reported that their greatest concern for oral health services in the future was the delay in completion of their dental treatment [95]. Patients undergoing orthodontic treatment were affected by lockdowns with some studies reporting that patients were anxious about resuming orthodontic treatments post lockdown [58, 113], concerns were also noted about outcomes due to the missed follow-up appointments [111].

## Discussion

This scoping review aimed to undertake a comprehensive mapping and synthesis of key findings related to the impact of the COVID-19 pandemic on oral health at the individual level. Scoping reviews do not exclude studies based on study design or quality appraisals which enables a synthesis of findings that provides a useful starting point for future reviews and other research activity [37, 38].

Government responses to the spread of COVID-19 varied across the world with many countries utilising lockdowns to stem the transmission. Service restrictions and lockdowns in many places resulted in limitation or cessation of essential services including dental services in some areas [17–20]. Our initial searches highlighted that much of the published dental and oral health research related to COVID-19 focused on the impact at the service level including risks for staff and financial impacts on dental practices. Whilst these issues are important at the professional level, we were interested in the impact that COVID-19 was having on oral health at the individual level.

Findings from this review were not surprising with many studies highlighting the impact that prolonged lockdowns had on dental treatment services (particularly emergency services) and routine oral health and preventive services. More recent studies have confirmed the impact that lockdowns had on dental service use by children, adults and older adults in the UK [14] with similar declines in paediatric services reported in Australia [31]. It is logical that closure of dental practices led to increased emergency visits and that many people would wait too long to seek care. Concerns have been raised that this could have resulted in more patients than usual requiring admission for the management of acute dental infections that can threaten airways and result in the need for intensive care [119].

Routine oral health care and preventive practices are cornerstones of good oral health across the lifespan. Reduction in these services throughout the lockdowns may have increased in oral healthcare-related disparities for vulnerable groups [14, 87, 120]. Changes to daily oral hygiene practices and home maintenance (including toothbrushing) were reported in this review (including increases and decreases). Twice daily toothbrushing with fluoride toothpaste continues to be recommended internationally to maintain oral hygiene with less frequent toothbrushing impacting overall oral health [121, 122]. Whilst changes in oral health behaviours were reported in the review the reasons for those changes were largely not reported. Stress and other disrupting events can affect the way people perceive their self-care needs and may impact people’s motivation to perform daily routines.

Changes in dietary preferences were reported in some studies with both increases and decreases in the consumption of sugary foods [97, 101]. High sugar consumption is a known risk factor for the development of dental caries [123]. A recent UK study showed that there were increases in reported purchases of confectionery, biscuits and sweet home cooking (all foods 'rich' in free sugars) among adults throughout the pandemic [14]. Whilst purchasing behaviours may not equate to consumption, high sugar consumption combined with reduced oral hygiene can lead to increases in dental disease.

The results of this review highlight the role that the internet played throughout the pandemic with many people using it for advice and/or information regarding oral health. Interruptions to dental services throughout the pandemic were challenging however access to quality information and evidence-based materials to support oral health care routines should be readily available. Help-seeking via the internet is common with previous studies highlighting using social media and search engines to find information about oral health is popular in some countries [124, 125]. However, concerns have been raised over the quality of the information provided by some sites [126]. The provision of quality information online would enable the continuation of daily oral health care routines throughout times of crisis and maybe something that dental practice, dental policymakers and other oral health-related service providers could consider in the future.

The use of teledentistry throughout the lockdown periods was reported within this review. Teledentistry may be useful to improve the ability to reach oral healthcare providers in times of crisis or to address service access issues [94], however further work is needed to explore the best models of provision (for example, prevention, consultations). In the absence of a face-to-face session with a dental professional, tele-dentistry may be a good option for oral health support and/or monitoring [45, 104]. However, teledentistry may not be available for those who do not have reliable access to technology and may increase inequalities for already vulnerable groups (rural, isolated).

Previous studies have highlighted that the widening inequalities in oral health cannot simply be “treated away” ([14]: p. 113) or addressed by increasing oral health care services. The pandemic has shown us that we need to reorient treatment systems to focus more on evidence-based systemic oral health improvement programmes like community water fluoridation, supervised toothbrushing programs, use of fluoride toothpaste to prevent the development of dental caries [14]. Whilst these activities are important, we also need to ensure that other oral health issues like periodontal disease and oral cancers that have significant economic costs [70] are included in our oral health improvement programmes in the future. Throughout the pandemic, we have also seen more emphasis on the role that oral and dental health professionals can play within the wider health system working more closely with other health providers in an integrated model of care [127]. More emphasis on these models will be central in future efforts to address oral health inequity and improve oral health across the globe [120, 127].

Many of the studies included in the review utilised cross-sectional, online, self-report surveys and existing datasets, with only one studyutilising qualitative methods. More qualitative studies could provide access to a deeper understanding of the factors that impact oral health at the individual level and could be used to inform the development of guidelines, policies and other information to improve oral health in the future [128, 129].

## Limitations

The searches that formed the basis of this review were carried out from March 2020 to November 2021. These dates were chosen to capture the majority of studies published while countries across the globe were under the highest level of restrictions (including lockdowns in many places). With the emergence of Omicron in November 2021, many countries lifted lockdowns as part of their approach to living with COVID-19. Whilst the searches were comprehensive, we have only captured those studies that were published in the included databases in a specific period. There may have been other studies published outside of these periods. We also limited the search to studies published in English with full-text availability which may have meant that some other studies were excluded.

As the emphasis of a scoping review is on comprehensive coverage and synthesis of the key findings, rather than on a particular standard of evidence we did not undertake a quality assessment of the included studies. This has resulted in the inclusion of a wide range of study designs and methodologies with small samples, cross-sectional designs using self-reported measures and internet-based research methods. The results of these studies provide valuable insights into the key issues faced by individuals throughout the pandemic however they must be interpreted with caution considering any specific methodological limitations.

Future research in this area should focus on addressing some of these key limitations with more research exploring the direct impacts of COVID-19 on individual oral health required. This will enable the development of specific oral health prevention activities that support good oral health through times of crisis.

## Conclusion

COVID-19 has impacted the oral health of many people across the globe, however, there are few reviews that have explored these impacts at the individual level. This review addresses this gap. The findings demonstrate that the pandemic has impacted the provision of emergency dental services, provision of routine oral health services, oral hygiene maintenance at home, changes in dietary preferences and the use of online information and help-seeking. It is important that we are aware of these impacts and ensure that support systems are in place to overcome future periods where access to dental care might be compromised. This will include ensuring patients have access to good quality oral health services and information including preventive care. A shift in focus that ensures a coordinated interdisciplinary approach consisting of treatment, education and prevention will reduce the pressure on emergency health care systems in the future.

## Supplementary Information

**Additional file 1**. PRISMA-ScR checklist

**Additional file 2**. Extracted data

## Data Availability

All data generated or analyzed during this study are included in this published article [and its supplementary information files].
